# Local Pixel Value Collection Algorithm for Spot Segmentation in Two-Dimensional Gel Electrophoresis Research

**DOI:** 10.1155/2007/89596

**Published:** 2007-09-27

**Authors:** Peter Peer, Luis Galo Corzo

**Affiliations:** ^1^CEIT and Tecnun (University of Navarra), Manuel de Lardizabal 15, 20018 San Sebastian, Spain; ^2^Faculty of Computer and Information Science, University of Ljubljana, Tržaška 25, 1000 Ljubljana, Slovenia

## Abstract

Two-dimensional gel-electrophoresis (2-DE) images show the expression levels of several hundreds of proteins where each protein is represented as a blob-shaped spot of grey level values. The spot detection, that is, the segmentation process has to be efficient as it is the first step in the gel processing. Such extraction of information is a very complex task. In this paper, we propose a novel spot detector that is basically a morphology-based method with the use of a seeded region growing as a central paradigm and which relies on the spot correlation information. The method is tested on our synthetic as well as on real gels with human samples from SWISS-2DPAGE (two-dimensional polyacrylamide gel electrophoresis) database. A comparison of results is done with a method called pixel value collection (PVC). Since our algorithm efficiently uses local spot information, segments the spot by collecting pixel values and its affinity with PVC, we named it local pixel value collection (LPVC). The results show that LPVC achieves similar segmentation results as PVC, but is much faster than PVC.

## 1. INTRODUCTION

Computer vision is a research line which tries to extract as much information from images as possible. Biomedical image analysis continues to be an active area of research, with many encouraging results, but also with a number of difficult problems still to be addressed [1].

Two-dimensional gel electrophoresis (2DE) is one of the methods able to separate thousands of proteins [2]. Different cell samples can exhibit even more than 2000 proteins. On such a 2D gel image, two coordinates characterize each protein: its isoelectric point and its molecular weight. Along one dimension, proteins are sorted electrophoretically according to their isoelectric point. They stabilize at points where their net charge is zero. Along the other dimension, proteins separate according to their molecular weight. Thus, the isoelectric point and the molecular weight uniquely identify a protein spot in a gel. The separated proteins can be stained with different dyes so that they are amenable to imaging. The gels are scanned and normally stored in a database. The process, though lengthy and subject to enormous experimental uncertainty, is still much cheaper than other competing technologies.

Figure 4(a) (neglect hand-marked annotations) shows a typical image of a 2D gel. Just by glancing at it, the reader can imagine how hard a task it is for any automated algorithm to accurately identify hundreds of protein spots among the various kinds of noise, and also to compare and match proteins over several gels when presented with multiple copies of gels made from similar cell samples.

There is a critical need for image analysis that will enable accurate, rapid and reliable spot detection [3]. The spot detection, that is, segmentation, process has to be efficient as it is the first step in the gel processing. Namely, inaccurate spot detection has clear ramifications for the spot matching process.

Before we go to the explanation of our algorithm, let us first take a look at the basic approaches to spot detection: Edge detection algorithms are traditionally used in such scenarios [4, 5]. Mathematical morphology-based methods are also widely used [6–8]. Popular methods include watersheds by immersion [9], marker-based watersheds [8], and H-domes method [7]. The scale space blob detection method can help us to select the markers [10, 11], which is seldom trivial. Our algorithm is basically a morphology-based method using seeded region growing as a central paradigm (see *The Image Processing Handbook* for standard algorithms [12]).

## 2. MATERIALS AND METHODS

### 2.1. Algorithm

In a recognition system, a preprocessing step to segment the pattern of interest from the background, noise, and so forth, usually precedes [13] the actual recognition process and for the current task there is no exception. 2DE images show the expression levels of several hundreds of proteins where each protein is represented as a blob shaped spot of grey level values. In our case, we are dealing with 8-bit grayscale images (while in the used SWISS-2DPAGE database [14] the images are available in 8-bit format), that is, 256 values are possible. In image processing, a common approach which speeds up a process is coarse-to-fine processing. In the context of gels this means that we could apply our methods first on 8-bit gel images and then refine obtained results on 16-bit images. Similary, we could first process smaller images and then refine the results on bigger images. The segmentation task at hand consists of a separation of the image into what is background and what are spots and the challenging parts are the cases of overlapping spots, varying background and a high level of noise in the images.

Namely, gel images are normally very noisy, so we first have to reduce the influence of noise on the subsequent processing, that is, to smooth the image. We do that by applying a 3×3 median filter [12] and then reducing the image size to the width of 500 pixels (with maintained aspect ratio), which also speeds up next steps of the algorithm. As already mentioned above, we could apply coarse-to-fine principle here to compensate for possible deleterious effects of the reduction step. Note that in SWISS-2DPAGE database [14], the images are stored with approximate width of 1000 pixels, but they are not preprocessed for noise and consequently we have to address the problem of noise. In the process of noise reduction, we conform to the rule that in any fitting or smoothing operation the window size has to be smaller than the features of interest [12]. Thus, in this preprocessing step, we reduce the noise and end up with more compact representation of spots.

**Figure 1:**
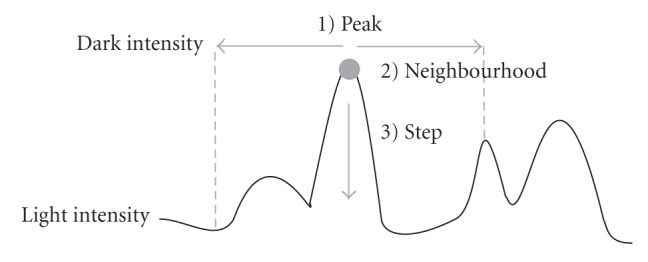
Basic principle of LPVC illustrated on a simple representation of an image intensity cross-section.

Since our algorithm efficiently uses local spot information and segments the spot by collecting pixel values, we named it local pixel value collection (LPVC). Figure 1 gives the basic principle of LPVC: find the peak (the darkest) value for each spot. For each peak locally in the semiuser-defined neighborhood, the spot grows to its boundaries by going through the local intensity range with the user-defined step. The pseudocode (see Algorithm 1) explains the algorithm in more detail as it gives its basic steps; note that we have two user-specified parameters: the initial number of the nearest neighbors used in the segmentation of each spot (NN) and the step size through the local intensity range (STEP).

The details about each step are given in the continuation.

The next step after preprocessing is to dynamically identify the background. This is achieved by applying a two-step Otsu thresholding technique [15]. The input to Otsu thresholding technique is a histogram of the input image, which is then divided in two classes and the interclass variance is minimized. Since a number of spots in the gel image are weakly expressed, we soften the border between the two classes, namely, spots and background, by applying the Otsu technique in two steps. First, we calculate the basic threshold and then this value is used to calculate the new, softened threshold based only on pixels in the image that are lighter than the calculated threshold. This dynamically obtained global threshold is then used to eliminate the background. (Note that we can apply this technique also locally on the image parts to better capture local properties of the background.) For more details about the technique see [15].

To identify spots, we interpret the intensity as the third-dimension information in the input image. We employ another operator in the 3×3 window size to identify local peaks. The peak is established if the pixel in the middle has the same or darker value as all surrounding, neighboring pixels. Generally, this operator is called 8-neighborhood filter [12].

Now that we have the information about peaks, we can correlate them in order to investigate spot sizes; but first we have to find the center of mass of each peak as they could be saturated, that is, a region bigger than one pixel can be labeled as peak. Normally, each spot is, among other information, represented by its x and y coordinate of the peak [16, 17]. In order to do this, we employ seeded region growing [12]. A seed can be the first pixel in the peak region and we recursively visit all the pixels in the peak region. In this way, we calculate for each peak its center of mass. For more details about the seeded region growing method see [12].

The first step towards establishing correlation of spots is to find the nearest neighbors for each identified peak. For this task, Euclidean distance [12] seems the most logical choice.

Now that we have this correlation information for each peak, we can eliminate some obvious nonpeaks based on the following condition: if the peaks are close together (we experimentally set this distance to d<6 pixels) and at the same time they have similar intensity values ($\Delta i_{\text{peaks}}<3$), while the intensity of the lightest pixel on the path between the peaks, is too similar to the intensity of the lightest peak ($\Delta i_{\text{path}}<3$), then we eliminate the lightest peak in a pair from further processing. The condition describes the fact that in such cases we are probably dealing with only one spot and not two.

As mentioned above, we have two user-specified parameters, which are used in the continuation of the algorithm: N is the initial number of the nearest neighbors used in the segmentation of each spot (NN in pseudocode) and S is the step size through the local intensity range (STEP in pseudocode).

In the next step, we again find $N_{\max}=8\;\left(N_{\max}\geq N\right)$ nearest neighbors for each kept peak.

**Figure 2:**
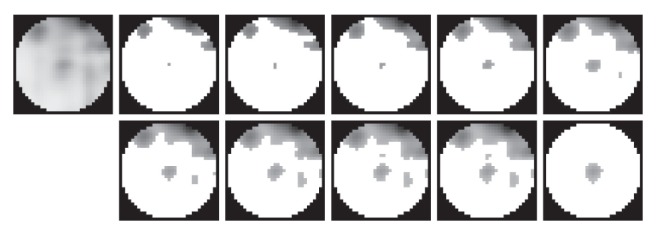
Illustration of LPVC segmentation of a single spot: in the circular region of interest we go through the local intensity range from the peak of interest intensity to the lightest intensity value, threshold the region of interest at each desired level, segment the temporary spot and test it if it is a real spot. The first figure gives the region of interest with the peak of interest centered in it and all in processing used local information. The last figure gives the last accepted spot by LPVC technique. All other figures illustrate which pixels are kept in the processing and how the spot of interest grows while we move through the intensity range.

For each kept peak we do the following using the preprocessed image (remember that this is the input image from which we eliminated noise): the region of interest is defined as an inner circle around the peak of interest with the radius defined by the truncated integer value of the distance to the Nth nearest neighbor (note the black mask in Figure 2). If all N−1 nearest neighboring peaks are outside of this region, then we increase N by one. This is repeated until at least one nearest neighbor is not inside the region of interest.

We find the lightest intensity value in the region of interest and we move inside the constrained intensity range from the peak of interest intensity to the lightest intensity value with step S (Figure 2). Firstly, we threshold the region of interest with the temporary intensity value. (For further details about thresholding see [12].) Secondly, we segment the temporary spot by applying the seeded region growing method, where the peak of interest is our seed. (For more details about the seeded region growing method see [12].) Thirdly, after the temporary spot is segmented, we check if it meets the criteria for the real spot. The criteria are the following: if there is a darker intensity value in the temporary spot than the peak intensity value, then we are not dealing with the real spot; we are not dealing with the real spot also if there is more than one peak kept in the temporary spot. Furthermore, if the number of pixels in the temporary spot is big enough (≥5), we check if it has a range of densities which peak centrally; if not, then we are not dealing with the real spot. Now, we check if it is approximately elliptical and if not, we are again not dealing with the real spot. The last criterion checks if the temporary spot size covers almost full region of interest; if so (≥80%), the temporary spot is not treated as the real spot. Thus, the temporary spot that meets all spot criteria is accepted as the real spot. Note that all the spot criteria except the first, the second, and the last one, which are specific for our algorithm, are also part of the algorithm in [6], with which we compare our results. The values are the same in both implementations. A short description of this approach is given in the continuation of the paper.

A speedup step is also added using the information about the actually present intensity values in the region of interest. Namely, the processing inside the region of interest is done only for intensity values that are represented in this region. Furthermore, if the value of step S is bigger than 1 and the temporary intensity value (threshold) is not present in the region of interest, then the nearest present and darker intensity value than the temporary threshold is taken as the new temporary threshold. Of course, we are always looking only inside the current interval of intensities defined by the step S. This speedup step is for more objective comparison, also added to the algorithm in [6], with which we compare our results.

When all the peaks are processed in this way, we end up with the segmented image and a linked list of information about each spot. As we will see in the next section, this information includes volume of each spot, which is one of the basic information used in the comparison of results.

The segmented image can now be superimposed over the original image with different degree of blending in order to help the user to focus on important parts of the gel for its subsequent processing (not shown). Blending can be implemented with a slider that blends the images based on the position of the handle on the slider, where each extreme of the slider represents one image, original and segmented. In this way, the segmentation results become even more intuitive for human perception.

### 2.2. Evaluation methodology

Unfortunately, we cannot simply count true positives (real spots), false positives, and so forth, in the real-gel images since the ground-truth information is not available. Moreover, when it comes to the human factor, such information is very subjective and varies even if the same person tries to provide this information at different occasions (e.g., try to mark the same image after one month and compare the markings). Furthermore, such counting would not be informative enough because it does not say anything about the segmentation accuracy of the individual spots.

To be as objective as possible, we evaluated the efficiency of LPVC technique in two steps. Since the ground-truth for real-gel images is not available, we first generated synthetic-gel images, which are generated based on desired ground-truth. In this way, the quantitative, numerical comparison is feasible. Then, we performed the experiments on real-gel images with human samples from SWISS-2DPAGE (two-dimensional polyacrylamide gel electrophoresis) database [14] (http://www.expasy.org/ch2d) in order to qualitatively, and visually evaluate the results.

Synthetic-gel images were generated by placing spots of a defined volume, size, and proximity in rows. Firstly, the image with the requested background value was generated (intensity =235). Secondly, each spot was modeled with a 2D Gaussian model. Thirdly, Gaussian noise was added to the image (standard deviation =2). For details about Gaussians please refer to [12, 18].

The first test addresses the precision of the technique by putting identical circular spots in the image (peak intensity =30, standard deviation =6). The second test addresses the intensity range by continuously lowering the peak height (intensity factor =0.9). In the third test, we continuously narrow the spot width. In terms of the mathematical model employed, we test the spot standard deviation range (standard deviation factor =0.9). The fourth test combines the last two together. In the last one, we continuously reduce the distance between spot pairs to simulate the proximity of spots (distance factor =0.8). This test is designed to enable assessment of the algorithm's ability to accurately split merged spots.

When we perform an experiment, we are basically interested in the values of two variables: the average error in the calculated spot volume and the time needed to process the whole image.

The spot volume is calculated in a standard manner [4]:

(1) $$\text{Vol}=\sum_{x\text{,}y\in \text{spot}}I\left(x,y\right),$$

where x and y are the coordinates of the pixel inside the spot and I(x,y) is the intensity value at these coordinates in the image.

The normalized error of the estimated spot volume Vol in comparison to the actual, ground-truth volume ${\text{Vol}}_{\text{GT}}$ (in % of ${\text{Vol}}_{\text{GT}}$) for the spot i is given as

(2) $${\text{Err}}_{\%\text{,}i}=\frac{\left|{\text{Vol}}_{i}-{\text{Vol}}_{\text{GT},i}\right|}{{\text{Vol}}_{\text{GT},i}}\cdot 100.$$

Furthermore, the average error ${\text{Avg}}_{\%}$ (arithmetic mean) over n spots present in the gel image is calculated. The second measure, which is in the results written right beside the first one (${\text{Avg}}_{\%}$), is the standard deviation, which reveals how tightly all the various estimated volumes are clustered around the average error in the set of data.

On real-gels, such quantitative evaluation is not feasible but the qualitative evaluation is. Thus, the influence of parameters on efficiency of the algorithm performance is investigated.

Finally, all the gels, synthetic and real, were also processed with a technique called pixel value collection (PVC) [6] for comparative performance. The pseudocode (see Algorithm 2) reveals the basic idea behind PVC and gives the affinity between LPVC and PVC.

Note that PVC always processes the whole image, while LPVC only the region of interest. PVC segments all spots at once at each level by applying a region labeling algorithm [6, 12], while LPVC employs a seeded region growing algorithm separately for each spot. Consequently, the merging of spots is treated differently in both approaches. For more details about PVC see [6]. Before going to the results, we should mention also the fact that in [6], a comparison of PVC with edge detection methodologies for spot detection is done. In discussion in [6], the authors state that PVC has potential advantages over known methods. The method is included in Phoretix 2D software from NonLinear Dynamics Ltd.

For an objective comparison of algorithms, the processing in both cases starts with the same preprocessing step described in the beginning of Section 2.1.

### 2.3. Time complexity

The time complexity of PVC is $O\left(n^{2}\right)$, where n gives the width and height of the processed image. Similarly, the time complexity of LPVC is $O\left(m^{2}\right)$, where m gives the width and height of the region of interest. Thus, in both cases we deal with squared time complexity, but since $m^{2}$ is much smaller in comparison with $n^{2}\;\left(m^{2}\ll n^{2}\right)$, the actual time needed to process the input image is much shorter for LPVC.

## 3. RESULTS AND DISCUSSION

### 3.1. Synthetic gels

**Figure 3:**
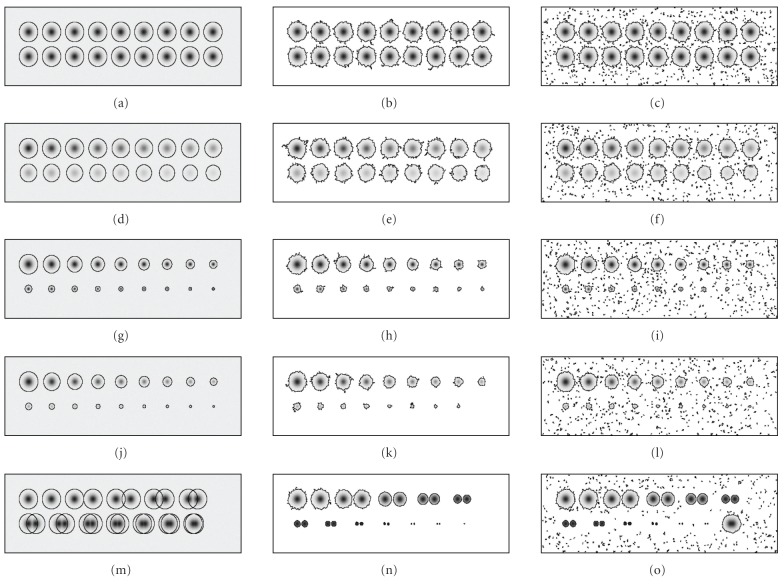
Computer-generated synthetic gels (first column; (a), (d), (g), (j), (m)) analyzed by proposed LPVC (second column; (b), (e), (h), (k), (n)) and PVC (third column; (c), (f), (i), (l), (o)). Gels are designed to demonstrate precision (first row; (a)–(c)), intensity range (second row; (d)–(f)), spot standard deviation range (third row; (g)–(i)), range in general (last two tests together) (fourth row; (j)–(l)), and effect of proximity of spots (fifth row; (m)–(o)). See Table 1 for quantitative evaluation and text for details.

Figure 3 presents the segmentation results of both algorithms applied to synthetic gels. The first column in Figure 3 gives the originals with correct ground-truth segmentation superimposed (panels (a), (d), (g), (j), (m)). The second column gives the segmentation results of the proposed LPVC technique ((b), (e), (h), (k), (n)), while the third one gives results of the PVC technique ((c), (f), (i), (l), (o)). In all segmentation results, spot areas are extracted from the original image array and transferred to a zero-ground array. The edge of segmented spots on synthetic gels is emphasized for better visualization. Remember that the gels contain the noise with the standard deviation of 1/3 of the standard deviation of the biggest spot in the gels. The step through the intensity range S was set in both algorithms to 1. The second parameter in LPVC, the initial number of nearest neighbors N used in the segmentation of each spot was also set to 1. In the first row we test precision ((a)–(c)), in the second intensity range ((d)–(f)), in the third spot standard deviation range ((g)–(i)), in the fourth we combine the last two ((j)–(l)), and in the fifth we test the effect of proximity of spots ((m)–(o)). (See Section 2.2 for details.)

By visually comparing the results, we can see that the segmentation of real spots is very similar, but PVC also finds nonreal spots at the lighter intensity range. On the other hand, Table 1 shows a moderate improvement of the LPVC average error of spot volume results (${\text{Avg}}_{\%}$) to PVC results and much faster segmentation of LPVC algorithm (t). (See Section 2.2 for details about the calculation of the average error.) In the last test (proximity), the average error is not calculated as it does not make sense: namely, both methods stop growing spot if more spots get merged. Thus, these spots do not reach their true boundaries. (A possible solution is pointed out in Section 4, where we discuss future work.)

As we will see in the continuation, the speedup achieved by LPVC is even more obvious when processing real-gels. Note also that the tests were performed on a single processor personal computer (Intel Pentium IV 3.0 GHz), in MS Visual Studio C++ debug mode.

The tests were also performed on gels without noise to see if the implementations of both algorithms are correct. In this case, both algorithms achieved optimal results (${\text{Avg}}_{\%}=0\pm 0\%$), while the ratio between times t remained similar.

### 3.2. Real-gels

Now, let us illustrate the performance of both algorithms on real-gels. (Remember that the ground-truth information for real-gels is not available.) We performed the experiments on real-gels with human samples from SWISS-2DPAGE database [14].

**Figure 4:**
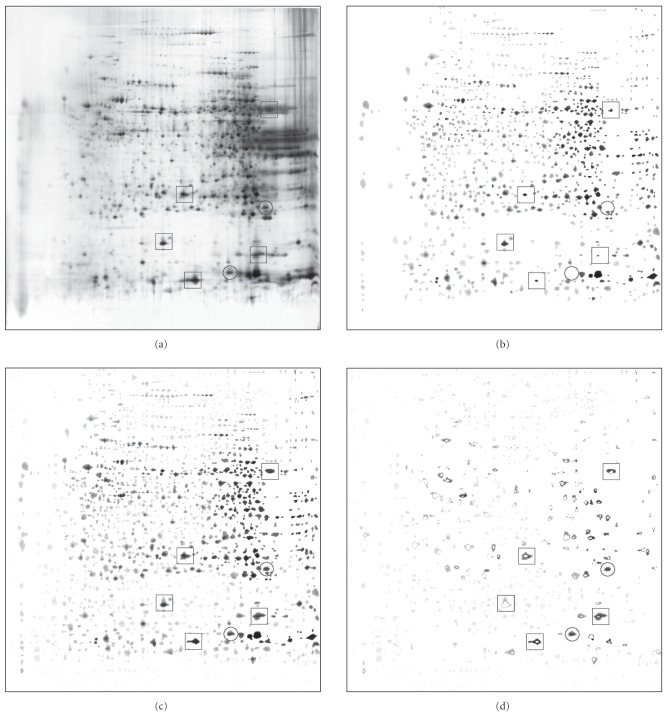
Segmentation results of LIVER gel from SWISS-2DPAGE database [14]: the original figure (a) is followed by LPVC result (6.6 seconds) (b), PVC result (17.9 minutes) (c), and the subtraction of PVC result from LPVC result (d). See text for details.

**Figure 5:**
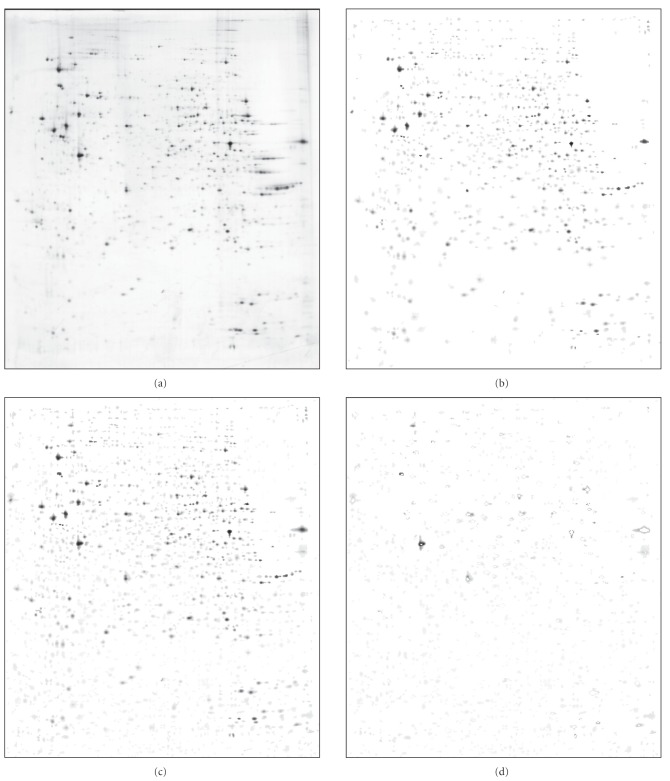
Segmentation results of U937 gel from SWISS-2DPAGE database [14]: the original figure (a) is followed by LPVC result (6.6 seconds) (b), PVC result (5.4 minutes) (c), and the subtraction of PVC result from LPVC result (d).

Figures 4 and 5 nicely illustrate the behavior of both algorithms. Figure 4 is LIVER gel from the database. It has many spots and is quite dark. On the other side, Figure 5 is U937 gel, which has much less spots and is much lighter. The processing was done with parameters set to: S=1, N=8. In both figures, we first give the original gel (panel a), then LPVC result (b), PVC result (c), and end with the subtraction of PVC result from LPVC result (d=b−c). The subtraction shows parts of spots that are present in PVC result and not in LPVC result. (The subtraction of LPVC result from PVC result is almost empty (not shown).)

From the results, we can see that the segmentations are very similar; but when we compare the times needed for the segmentation, we see that LPVC is much faster: while PVC for the segmentation of LIVER gel (Figure 4) needs 1073.3 seconds (≈17.9 minutes), LPVC needs only 6.6 seconds. For the segmentation of U937 gel (Figure 5), PVC needs 325 seconds (≈5.4 minutes) and LPVC needs only 6.6 seconds.

In Figure 4 we hand marked a few spots with squares and circles to point out a few properties of both segmentation techniques. Let us first focus on squares: based on spots like these, it is most probable that PVC in general is more prone to over-segmentation than LPVC. The properties marked with circles are even more interesting. They denote spots that are detected by PVC and not by LPVC. The reason for this can be found in the different approaches to segmentation. Namely, in the case of the lower circle, LPVC stops growing the spot when a decision of nonspot is reached, while PVC tries again on the next intensity level. In the case of the upper circle, the segmented spot by PVC actually merges two spots together in one, while LPVC rejects both because they are too small to be treated as real spots.

But LPVC can segment the spots like the one inside the lower circle simply by stopping the growing after two successive decisions of nonspot are reached. Furthermore, LPVC can accept the two spots inside the upper circle simply if we lower the minimal required spot size. Thus, integration of such options into the software actually gives better expected results (not shown). Consequently, the time needed to process a gel is a bit longer, but LPVC is still much faster than PVC.

### 3.3. Influence of parameters

In this section we demonstrate the influence of parameters on the segmentation process. For the step S through the intensity range, it is obvious that with bigger S we make a compromise between the accuracy and speed. With bigger S we achieve faster execution, but lower accuracy. To illustrate the achieved speedup for S=10 on LIVER gel, we give the times needed to process the gel with both algorithms: LPVC – 3 seconds, PVC – 107.4 seconds (≈1.8 minutes). Thus, LPVC with S=1 is still much faster than PVC with S=10 and, of course, achieves better accuracy.

**Figure 6:**
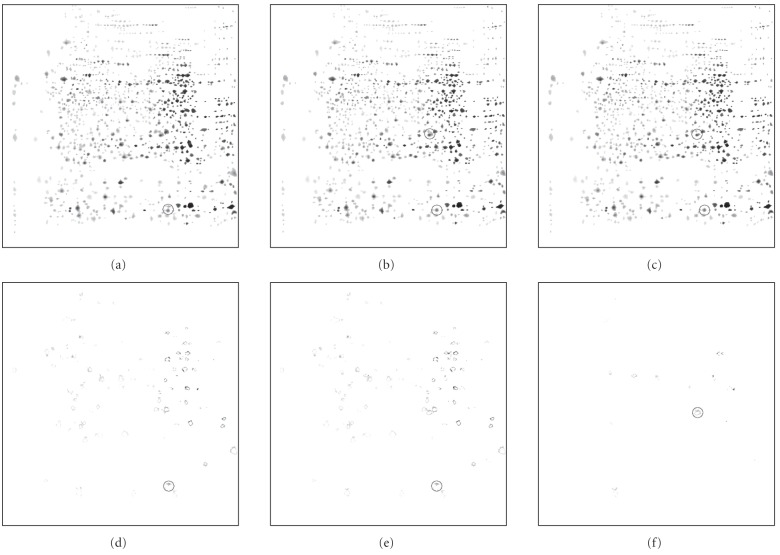
Comparison of results obtained with LPVC for different values of the initial number of nearest neighbors N used in the segmentation of each spot (LIVER gel [14]). The first result was obtained with N=1 (3.1 seconds) (a), the second with N=5 (4.7 seconds) (b), and the third one with N=8 (6.6 seconds) (c). The second row gives differences between obtained results (d)–(f). See text for details.

Figure 6(a) shows a comparison of results obtained with LPVC for different values of the initial number of nearest neighbors N used in the segmentation of each spot (S=1). (Remember that PVC does not have this parameter.) The original gel can be seen in Figure 4(a) (LIVER gel). The first result was obtained with N=1 (3.1 seconds) (see Figure 6(a)), the second with N=5 (4.7 seconds) (panel b) and the third one with N=8 (6.6 seconds) (c). The second row gives differences between obtained results: the difference between the second result and the first (panel d=|b−a|), then the difference between the third result and the first (e=|c−a|), and finally, the difference between the third result and the second (f=|c−b|). The differences show parts of spots that are present only in one of the compared results.

The main conclusion that can be drawn from Figure 6 is that with bigger N, we achieve better segmentation (see hand marked circles), while the time needed for the segmentation does not increase substantially. We can also observe that by using N=5, we achieve a good compromise between time and accuracy. But since the execution is fast even with N=8, we are not forced to make this compromise.

## 4. CONCLUDING REMARKS

This paper presents a novel algorithm called local pixel value collection, a sequence of steps which leads to the spot segmentation of 2DE images. LPVC similarly to PVC, to which we contrasted LPVC results, builds on morphology idea, but in contrast to PVC extensively uses local spot information. Thus, LPVC achieves similar segmentation results as PVC much faster. In its current format, once segmented, the resultant image is suitable for registration and comparison processes typical of 2DE image analysis workflows. Whilst this approach will not resolve all of the issues surrounding the major bottleneck in 2DE gel-based proteomic analysis, it gives us a good starting point for future work and the subsequent processing. The fact is that its results help the user to focus on important parts of the gel.

Because of possible proximity of spots to each other, we have to grow such spots to their real borders (see the last row, panels (m)–(o) in Figure 3). This could, for instance, be addressed by parametric spot modeling with Gaussian, diffusion or mixture spot model [18, 19]. This task is the first one to be addressed in our future work.

LPVC approach will be included as one of the options in our 2D gel analysis software that we are developing.

## Figures and Tables

**Algorithm 1:**
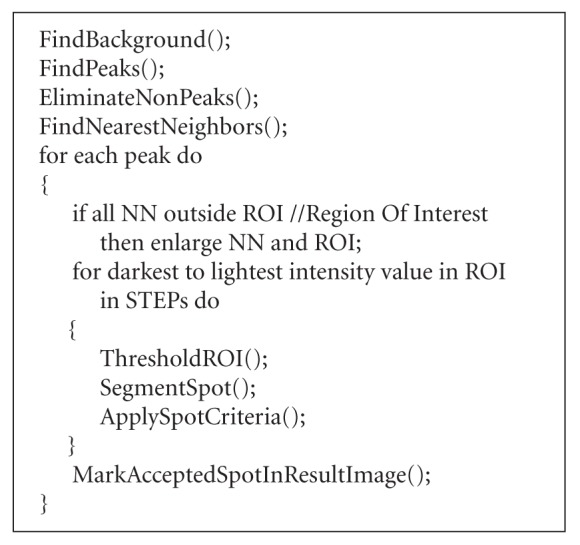


**Algorithm 2:**
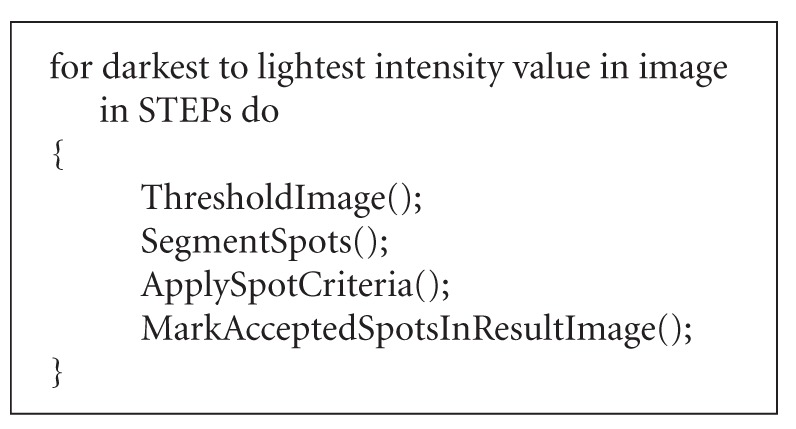


**Table 1:** Evaluation of the proposed LPVC method on computer-generated synthetic gels and comparison of results with the PVC method (see Figure 3): t gives the time in seconds needed to segment the gel, and ${\text{Avg}}_{\%}$ gives the normalized average error of segmented spot volumes in percentage of correct, ground-truth spot volumes and its standard deviation. Smaller the values, better the results. See text for details.

Test	LPVC		PVC	
	t[s]	Avg_%_ [%]	t[s]	Avg_%_ [%]
Precision	9.5	0.6 ± 0.4	31.7	1 ± 1.5
Intensity range	3.6	1.5 ± 1	13.7	4.8 ± 6.2
St. dev. range	7	2.6 ± 2.8	13.7	3 ± 2.9
Both ranges	2.7	9 ± 11.9	9.2	9.5 ± 11.5
Proximity	3.1	—	32.4	—
